# Construction and validation of a risk model based on the key SNARE proteins to predict the prognosis and immune microenvironment of gliomas

**DOI:** 10.3389/fnmol.2023.1304224

**Published:** 2023-12-05

**Authors:** Luxin Yin, Yiqiang Xu, Jiale Yin, Hai Cheng, Weihan Xiao, Yue Wu, Daofei Ji, Shangfeng Gao

**Affiliations:** ^1^Department of Neurosurgery, The Affiliated Hospital of Xuzhou Medical University, Xuzhou, China; ^2^Institute of Nervous System Diseases, Xuzhou Medical University, Xuzhou, China; ^3^Department of Neurosurgery, The Second Affiliated Hospital of Xuzhou Medical University, Xuzhou, China

**Keywords:** glioblastoma, VAMP2, VAMP5, immune infiltration, bioinformatics

## Abstract

**Background:**

Synaptic transmission between neurons and glioma cells can promote glioma progression. The soluble N-ethylmaleimide-sensitive fusion factor attachment protein receptors (SNARE) play a key role in synaptic functions. We aimed to construct and validate a novel model based on the SNARE proteins to predict the prognosis and immune microenvironment of glioma.

**Methods:**

Differential expression analysis and COX regression analysis were used to identify key SRGs in glioma datasets, and we constructed a prognostic risk model based on the key SRGs. The prognostic value and predictive performance of the model were assessed in The Cancer Genome Atlas (TCGA) and Chinese glioma Genome Atlas (CGGA) datasets. Functional enrichment analysis and immune-related evaluation were employed to reveal the association of risk scores with tumor progression and microenvironment. A prognostic nomogram containing the risk score was established and assessed by calibration curves and time-dependent receiver operating characteristic curves. We verified the changes of the key SRGs in glioma specimens and cells by real-time quantitative PCR and Western blot analyses.

**Results:**

Vesicle-associated membrane protein 2 (VAMP2) and vesicle-associated membrane protein 5 (VAMP5) were identified as two SRGs affecting the prognoses of glioma patients. High-risk patients characterized by higher VAMP5 and lower VAMP2 expression had a worse prognosis. Higher risk scores were associated with older age, higher tumor grades, IDH wild-type, and 1p19q non-codeletion. The SRGs risk model showed an excellent predictive performance in predicting the prognosis in TCGA and CGGA datasets. Differentially expressed genes between low- and high-risk groups were mainly enriched in the pathways related to immune infiltration, tumor metastasis, and neuronal activity. Immune score, stromal score, estimate score, tumor mutational burden, and expression of checkpoint genes were positively correlated with risk scores. The nomogram containing the risk score showed good performance in predicting the prognosis of glioma. Low VAMP2 and high VAMP5 were found in different grades of glioma specimens and cell lines.

**Conclusion:**

We constructed and validated a novel risk model based on the expression of VAMP2 and VAMP5 by bioinformatics analysis and experimental confirmation. This model might be helpful for clinically predicting the prognosis and response to immunotherapy of glioma patients.

## 1 Introduction

Gliomas are the most common primary malignant brain tumors in the central nervous system (Yang et al., 2022). The latest World Health Organization (WHO) classification criteria divide gliomas into three categories, based on their histopathological and molecular characteristics, i.e., oligodendrogliomas with IDH mutations and chromosome 1p19q co-deletions, astrocytomas with IDH mutations, and glioblastomas with wild-type IDH (Choi et al., 2019). In spite of the current multimodal treatment approach for gliomas, including surgery, radiotherapy, chemotherapy, and electric field therapy, the prognosis remains poor, with almost all patients experiencing tumor progression or recurrence (Barthel et al., 2019). Previous studies have demonstrated that several molecular markers, such as IDH mutations, chromosome 1p/19q co-deletions, etc., can be used for glioma diagnosis, treatment planning and prognosis assessment (Eckel-Passow et al., 2020). However, targeted therapy for these molecular markers has not achieved obvious results in clinical practice. To improve the prognosis of gliomas, it is essential to identify new biomarkers that may lead to more precise and personalized treatments.

In addition to intrinsic cellular mechanisms, tumor microenvironment plays a crucial role in glioma progression. Neurons in the glioma microenvironment regulates the malignant growth of gliomas in an activity-dependent manner (Venkatesh et al., 2019). The hyperexcitability of neurons in gliomas leads to increased activity of glutamatergic neurons, which causes glioma growth and glioma-associated seizures (Gillespie and Monje, 2018). The SNARE proteins are involved in many essential neuronal functions, such as neuronal growth, axonal remodeling, axonal extension, synaptogenesis, and synaptic transmission (Li and Kavalali, 2017). The main SNARE protein complex is composed of VAMP2, synaptosomal-associated protein 25 (SNAP-25), and syntaxin-1 (syn1) (Heo et al., 2016). It has been reported that SNARE protein-related genes (SRGs) play a role in several types of cancer, such as lung cancer, breast cancer, and digestive tumors (Nan et al., 2018; Chen et al., 2021; Mitani et al., 2022). However, the specific role of SNARE proteins in glioma is still poorly studied.

In the current study, we attempted to evaluate the prognostic value of SRGs in glioma patients by bioinformatics analyses followed by experimental confirmation. RNA-seq data and clinical characteristics of glioma patients were obtained from public databases. We examined the relationship between SRGs and clinicopathological factors in glioma patients. We constructed a risk model using key SRGs, and analyzed the differences in prognosis, clinicopathological features, and immune infiltration between low- and high-risk patients. More importantly, we verified the changes of VAMP2 and VAMP5 at both mRNA and protein levels in glioma specimens and cell lines.

## 2 Materials and methods

### 2.1 Datasets and processing

We downloaded RNA-seq data and clinicopathological information from TCGA,^1^ NCBI GEO^2^ and CGGA.^3^ For the data from TCGA and GEO databases, RNA-seq count values were used for differential expression analysis, while Transcripts Per Million (TPM) values was employed for survival analysis. For the data from CGGA325 and CGGA301 datasets, Fragments Per Kilobase Million (FPKM) values were used for both expression and survival analyses. The TCGA, GSE147352, and CGGA datasets were normalized separately. Patients with incomplete prognostic details were excluded.

### 2.2 Identification of key SNARE protein-related genes

The TCGA dataset contains 169 glioblastoma samples, 529 low-grade glioma samples, and 5 normal tissue samples. The GSE147352 dataset contains 15 non-tumor samples and 103 tumor samples (18 low-grade gliomas, 85 glioblastomas). In the TCGA and GSE147352 datasets, differentially expressed genes (DEGs) in the glioma and non-tumor samples were identified using the DESeq2 R package (DESeq2) and the limma package. Thresholds were set at *P* adj < 0.05 and absolute logFC values >1. We collated 36 SNARE protein-associated genes based on published literature in the PubMed database. The overlapping DEGs were screened using Venn diagram. By analyzing the differential genes in glioma tissue and normal tissue in TCGA and GSE147352 data sets, we obtained the common differential genes between the two datasets, and then intersected with 36 SNARE protein-related genes to obtain a total of 6 DEGs.

### 2.3 Construction of a risk model based on SNARE protein-related genes

The univariate and multivariate Cox regression analyses were performed on six overlapping DEGs. Genes with a *P*-value < 0.05 were considered significant prognostic values. Using those genes, we built a SRGs risk model for predicting overall survival (OS) with the following equation: Risk score = gene 1 expression × (b1) + gene 2 expression × (b2) + gene n expression × (bn); b represents the multifactor Cox regression coefficient. Patients were divided into high-risk and low-risk groups according to the median risk score. Kaplan-Meier survival analysis was performed to determine the prognostic difference between low-risk and high-risk patients. Receiver operating characteristic (ROC) curves were plotted using the timeROC R package to assess the accuracy of the SRGs risk model in predicting patient survival outcomes.

### 2.4 Construction of a prognostic nomogram

In order to determine whether SRGs risk score can serve as an independent prognostic factor, we included age, sex, WHO classification, IDH mutation status, and 1p19q coding deletion status for univariate and multivariate Cox regression analyses. According to the Cox proportional risk test and multivariate Cox regression, a nomogram was constructed using the rms R package. Calibration curve and ROC curve were used to evaluate the prediction performance of the nomogram. The nomogram is also externally validated in the CGGA325 and CGGA301 cohorts.

### 2.5 Bioinformatic analysis based on the SRGs risk model

We used the “DESeq2” R package to identify DEGs between high-risk and low-risk groups. A threshold was set at *P* < 0.05. In order to explore the potential biological pathways and functions of the high-risk and low-risk groups, enrichment analysis was performed on DEGs. Gene Ontology (GO) analysis and Kyoto Encyclopedia of Genes and Genomes (KEGG) functional enrichment analysis were performed on high and low risk group DEGs using the clusterprofiler R package. The REACTOME gene set was downloaded from the Molecular Signature Database (MSigDB) for Gene Set Enrichment Analysis (GSEA) enrichment analysis to explore the underlying molecular mechanisms in the high and low risk groups, and a false discovery rate (FDR) <0.05 was considered statistically significant.

### 2.6 Evaluation of immune microenvironment based on the SRGs risk model

The “ESTIMATE” package in R software was used to calculate the stromal score, immune score, estimate score and tumor purity for each sample in the TCGA glioma dataset. We used the “CIBERSORT” algorithm to calculate the relative infiltration levels of 22 immune cells in high-risk and low-risk group. The Wilcoxon signed rank test was used to compare immune infiltration levels between low- and high-risk patients. In each sample, the maximum enrichment score for all immune cell types was 1. We analyzed the relationship between SGRs risk scores and the expression levels of 13 clinically common immune checkpoint genes using Spearman’s rank correlation test. Tumor Mutation Burden (TMB) data was downloaded from TCGA and compare between high- and low-risk groups.

### 2.7 Patients and samples

All normal tissue and glioma tissue specimens used for validation were obtained from the Affiliated Hospital of Xuzhou Medical University. Normal brain tissue specimens were obtained from brain tissue removed during traumatic brain injury surgery. All glioma patients did not receive immunotherapy, radiotherapy or chemotherapy. Fresh samples were stored at −80°C immediately after surgical resection. A total of 35 samples (including 9 normal tissues and 24 glioma tissues of different grades) were included in the qRT-PCR analysis, and 32 samples (including 8 normal tissues and 24 glioma tissues of different grades) were analyzed by Western blot. All glioma specimens had clear pathological diagnoses and IDH1 status. They were classified according to the WHO (2021) classification of central nervous system tumors (Louis et al., 2021). The clinicopathological information of all subjects was provided in Supplementary Table 1.

### 2.8 Cell lines and cell culture

Glioma cell lines (U87, U251, A172, U373, and LN229) were purchased from the Shanghai Cell Bank, Type Culture Collection Committee, Chinese Academy of Sciences (Shanghai, China). Human astrocyte line HA1800 was generously gifted by Dr. Jiantong Jiao, as mentioned in our previous publication (Meng et al., 2021). U87 were cultured in Minimal Essential Medium (Gibco, Grand Isle, NY, USA) and the rest of cell lines were grown in Dulbecco’s Minimal Eagle’s Medium (Bio-Channel Biotechnology, Nanjing, China), supplemented with 10% fetal bovine serum (FBS, Gibco).

### 2.9 Western blot analysis

A standard protocol was followed to extract total proteins from tissues and cell lines (Cheng et al., 2008). Protein concentrations were determined using the BCA protein assay kit (Beyotime Biotechnology, Shanghai, China). We performed Western blotting as described in our recent publication (Meng et al., 2021). The tissue samples were loaded in the order described in Supplementary Table 2. The primary antibodies used for immunoblotting were as follows: anti-VAMP2 (Proteintech, 10135-1-AP, 1:2000), anti-VAMP5 (Proteintech, 11822-1-AP, 1:1000), and anti-GAPDH (Proteintech, 10491-1-AP, 1:10000), β-actin (Proteintech, HRP-66009, 1:40000). We used ImageJ software (National Institutes of Health, Bethesda, MD, USA) to quantify band densities. Relative protein levels were determined by normalizing the densitometric values of the protein of interest to those of GAPDH.

### 2.10 RNA extraction and quantitative qRT-PCR

Total RNA extraction and reverse transcription were performed as described in our previous paper (Zhang et al., 2018). The target genes were amplified using ChamQ SYBR Color qPCR Master Mix (Vazyme, Nanjing, China) and a mixture of forward and reverse primers in a final volume of 20 μL as follows: VAMP2 (forward: 5′-ACATTACTCGCAGGTGGTGG-3′, reverse: 5′- GAGAAACCCGAAGTGGCAGA-3′); VAMP5 (forward: 5′-AGCGTTCAGACCAACTCCTG-3′, reverse: 5′-GATGAGCAGGACCAACCA-3′), and GAPDH (forward: 5′-ACCACAGTCCATGCCATCAC-3′, reverse: 5 ′-TCCACCACCCTGTTGCTGTA-3). The same PCR temperature cycle and real-time PCR system was used as previously described (Meng et al., 2021). Data were automatically acquired and processed using Applied Biosystems 7500 (Applied Biosystems, Foster City, CA, USA). The expression of VAMP2, VAMP5 was normalized to that of GAPDH, and the relative absolute expression of VAMP2, VAMP5 was calculated according to our previous statistical methods (Gao et al., 2013).

### 2.11 Statistical analysis

Bioinformatics analysis was performed in R software. As not all data were distributed normally, Wilcoxon rank sum test was conducted to compare the differences between two groups, Kruskal-Wallis test followed by Dunn’s test was used to compare the differences in three or more groups. All survival analysis was performed using the multifactorial COX regression model, Kaplan-Meier curve and Log-Rank test. Spearman’s rank test was used for correlation analysis. We used a *P*-value < 0.05 to be statistically significant.

## 3 Results

### 3.1 Identification of SNARE family signature genes in glioma

**FIGURE 1:**
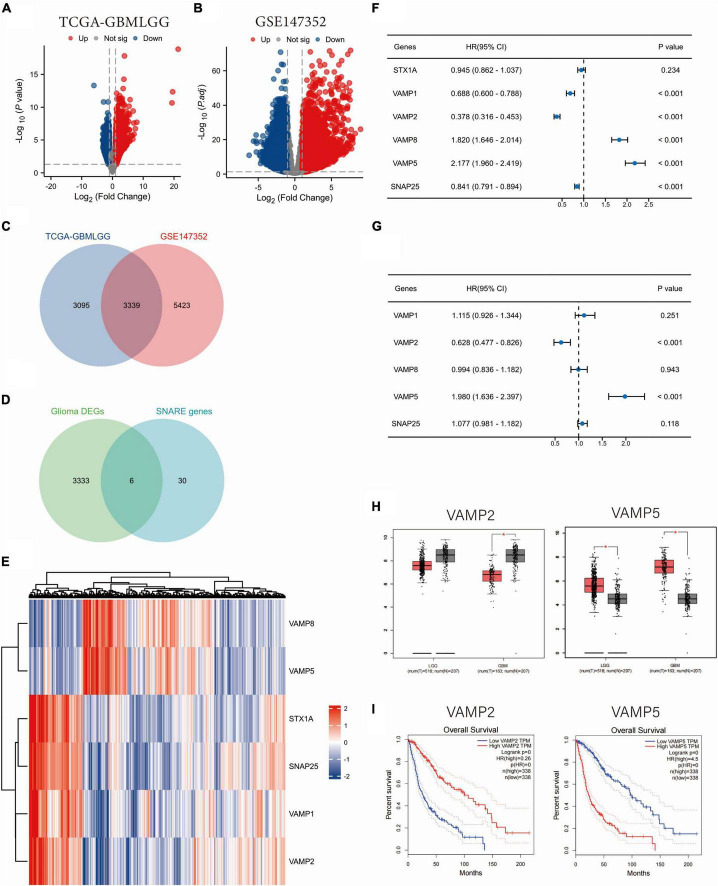
Identification of SNARE family signature genes in glioma. **(A,B)** Volcano maps of DEGs between glioma and normal samples. Data were downloaded from TCGA **(A)** and GSE147352 **(B)** databases. **(C)** Venn diagram showing the overlapping DEGs between the two datasets. **(D)** Venn diagram showing the DEGs in SNARE family. **(E)** Heat map showing the expression profile of the 6 SNARE family genes in the TCGA dataset. Univariate **(F)** and multivariate **(G)** Cox regression analyses of the six SNARE family genes in relation to patients’ prognosis. **(H)** The expression changes of VAMP2 and VAMP5 between LGG/GBM and normal samples. **(I)** The survival analysis of VAMP2 and VAMP5 in glioma. (*p < 0.05).

By analyzing the TCGA and GSE147352 datasets, 6434 and 8762 DEGs were identified between glioma and normal tissues, respectively (Figures 1A, B). There are 3339 DEGs overlapping between the two datasets. A total of 6 SRGs (intersection of 3339 DEGs and 36 SRGs) were screened by Venn analysis (Figures 1C, D). Two of the 6 SRGs were up-regulated and 4 were down-regulated in glioma tissues (Figure 1E). Univariate Cox regression analysis showed that 5 SRGs (VAMP1, VAMP2, VAMP5, VAMP8, and SNAP25) were significantly associated with patients’ over survival (OS) in the TCGA dataset (Figure 1F). We then performed a multivariate Cox regression analysis, and the results showed that two SRGs (VAMP2 and VAMP5) had significant prognostic value in gliomas (Figure 1G). According to the Gene Expression Profile Interaction Analysis (GEPIA) online analysis, VAMP5 expression was significantly higher, while VAMP2 expression was significantly lower in gliomas than in normal samples (Figure 1H). Survival analysis showed that patients with high VAMP5 expression had a poorer prognosis than patients with low expression, whereas patients with high VAMP2 expression had a better prognosis than patients with low expression (Figure 1I). These data suggested that VAMP2 and VMAP5 were the key genes in the SNARE family that affected the prognosis of glioma patients.

### 3.2 Construction and validation of a risk model based on the expression of VAMP2 and VAMP5

**FIGURE 2:**
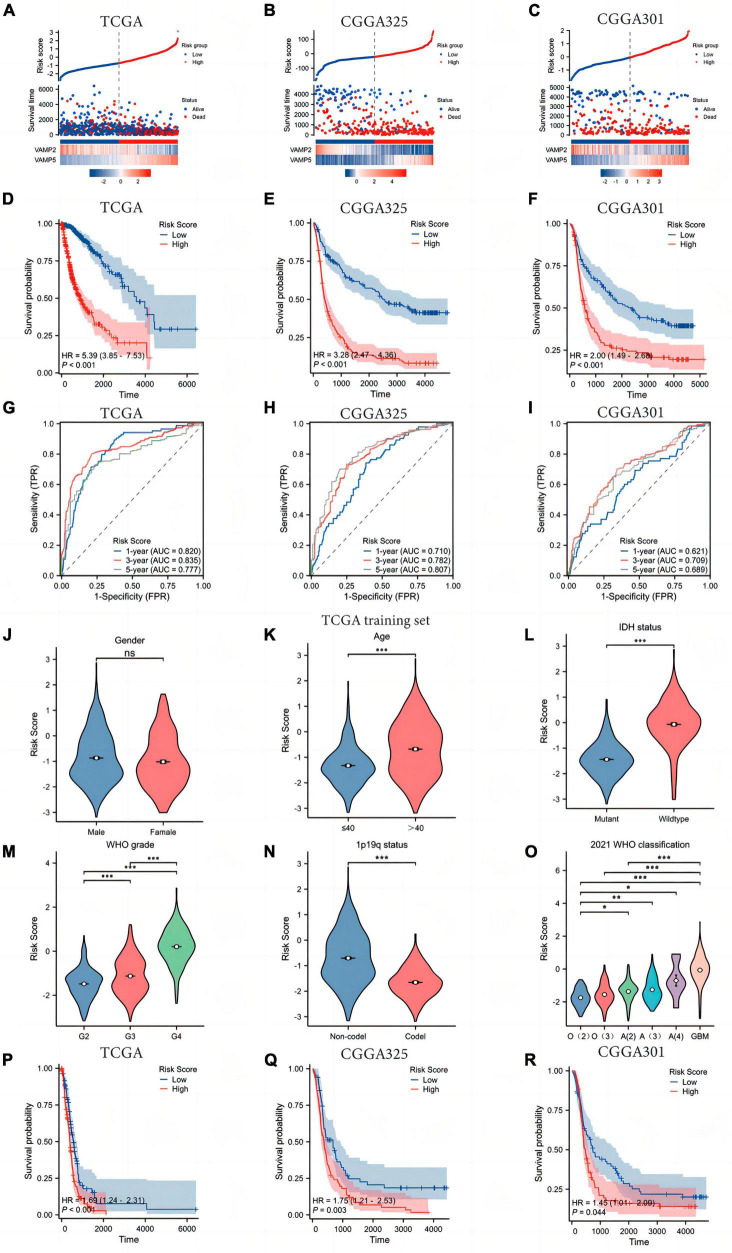
Construction and validation of the SRGs risk model. **(A–C)** The survival distribution and expression profiles of VAMP2 and VAMP5 in low- and high-risk patients. **(D,E)** The Kaplan-Meier plot showing the difference in overall survival (OS) between low- and high-risk groups. **(G–I)** The time-dependent ROC curve was plotted to assess the SRGs risk model in predicting 1 -, 3 -, and 5-year OS of glioma patients. **(J–O)** The associations between the risk score and clinicopathological features. O(2): Oligodendroglioma WHO 2 grade; O(3): Oligodendroglioma WHO 3 grade; A(2): Astrocytoma WHO 2 grade; A(3): Astrocytoma WHO 3 grade; A(4): Astrocytoma WHO 4 grade; GBM: Glioblastoma WHO 4 grade. **(P–R)** Survival analysis between low- and high-risk patients in IDH wild-type gliomas. (*p < 0.05; **p < 0.01; ***p < 0.001; ns, not significant).

The two key SRGs obtained from the above analysis were used to construct a prognostic risk model according to the formula as follows: Risk score = VAMP5 expression level × 0.683-VAMP2 expression level × 0.465–1.9625 (Figure 2A). Patients were divided into low- and high-risk groups based on the median of risk scores. High-risk patients characterized by higher VAMP5 and lower VAMP2 expression, had a worse prognosis than low-risk patients in the TCGA dataset (Figures 2A–C). Survival analysis further exhibited that patients in high-risk group had significantly shorter OS than those in low-risk group (HR = 5.39, 95% CI = 3.85–7.53, Figure 2D). Similar results were found in the CGGA325 (HR = 3.28, 95% CI = 2.47–4.36) and CGGA301 (HR = 2.00, 95% CI = 1.49–2.68) datasets (Figures 2E, F). We conducted a time-independent ROC curve analysis to assess the specificity and sensitivity of the SRGs risk model in glioma patients. The area under the curve (AUC) for the TCGA dataset was 0.820, 0.835, and 0.777 at 1, 3, and 5 years, respectively. The AUC for the CGGA325 dataset was 0.710, 0.782, and 0.807 at 1, 3, and 5 years, respectively. The AUC for the CGGA301 validation dataset was 0.621, 0.709 and 0.689 at 1, 3 and 5 years, respectively (Figures 2G–I). These results suggested that the SRGs risk model had an excellent predictive performance in predicting the prognosis of glioma patients. In addition, we found significant differences in the risk scores in patients with different clinicopathological characteristics in the TCGA dataset. High risk scores were positively associated with older age, higher WHO grade, IDH wild-type status, and 1p19q non-codeletion, but not with gender (Figures 2J–N). Notably, our model could distinguish well among gliomas of different molecular subtypes classified by the WHO (2021) CNS classification criteria (Figure 2O). Interestingly, when we performed survival analysis in IDH wild-type glioma patients, there was also significant differences in the OS between patients with low- and high-risk scores (Figures 2P–R). We found the similar results in the CGGA325 and CGGA301 datasets (Supplementary Figure 1).

### 3.3 Enrichment analysis of the DEGs between low- and high-risk groups

**FIGURE 3:**
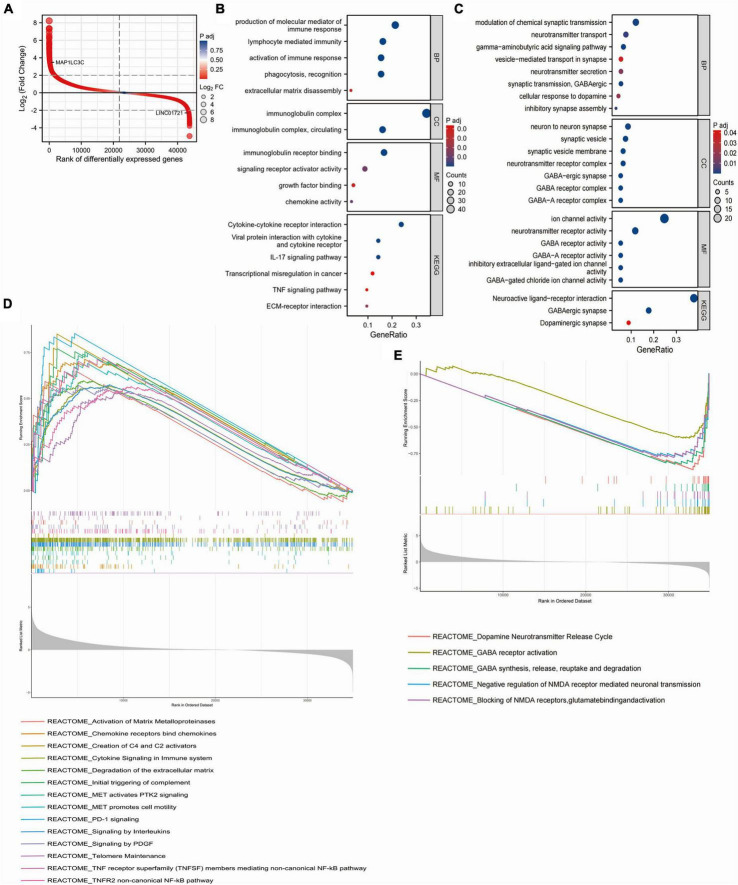
Enrichment analysis of DEGs between high- and low-risk groups. **(A)** Differential sequencing map of DEGs between high and low risk groups. **(B)** biological process (BP), molecular function (MF), cell component (CC) and Kyoto Encyclopedia of Genes and Genomes (KEGG) enrichment analysis of the top 200 DEGs highly expressed in the high-risk group. **(C)** biological process (BP), molecular function (MF), cell component (CC) and Kyoto Encyclopedia of Genes and Genomes (KEGG) enrichment analysis of the top 200 DEGs highly expressed in the low-risk group. **(D)** GSEA results of DEGs with higher expression in the high-risk group. **(E)** GSEA results of DEGs with higher expression in the low-risk group.

We screened DEGs between high- and low-risk groups in the TCGA glioma dataset, and the top 200 highly expressed genes in the high-risk group (MAP1LC3C was the 200th gene) and the top 200 highly expressed genes in the low-risk group (LINC01721 was the 200th gene) (Figure 3A) were used for GO and KEGG enrichment analyses. The results showed that the top 200 highly expressed genes in the high-risk group were mainly enriched in pathways that promote immune infiltration, tumor proliferation and metastasis, such as production of molecular mediators of immune response, extracellular matrix (ECM) breakdown, cytokine-cytokine receptor interaction, ECM-receptor interaction, etc. (Figure 3B). In contrast, the high-expressed genes in the low-risk group were mainly enriched in the modulation of chemical synaptic transmission, vesicle-mediated transport in synapses, neurotransmitter secretion, neuroactive ligand-receptor interactions, and other pathways that inhibit neuronal activity (Figure 3C). GSEA analysis showed that genes highly expressed in the high-risk group were mainly enriched in pathways related to tumor metastasis and immune invasion, including activation of matrix metalloproteinases, chemokine receptors bind chemokines, cytokine signaling in immune system, programmed death 1 (PD-1) signaling, and telomere maintenance (Figure 3D). In contrast, genes highly expressed in the low-risk group were mainly enriched in molecular pathways that inhibit neuronal activity, mainly including dopamine neurotransmitter release cycle, Gamma-aminobutyric (GABA) synthesis, release, reuptake and degradation, negative regulation of *N*-methyl-D-aspartate (NMDA) receptor-mediated neuronal transmission, and glutamate binding and activation (Figure 3E). These results suggested that there were significant differences in the tumor immune, tumor proliferation and metastasis, and neuronal activity between low- and high-risk patients.

### 3.4 Analysis of glioma immunological features using the SRGs risk model

**FIGURE 4:**
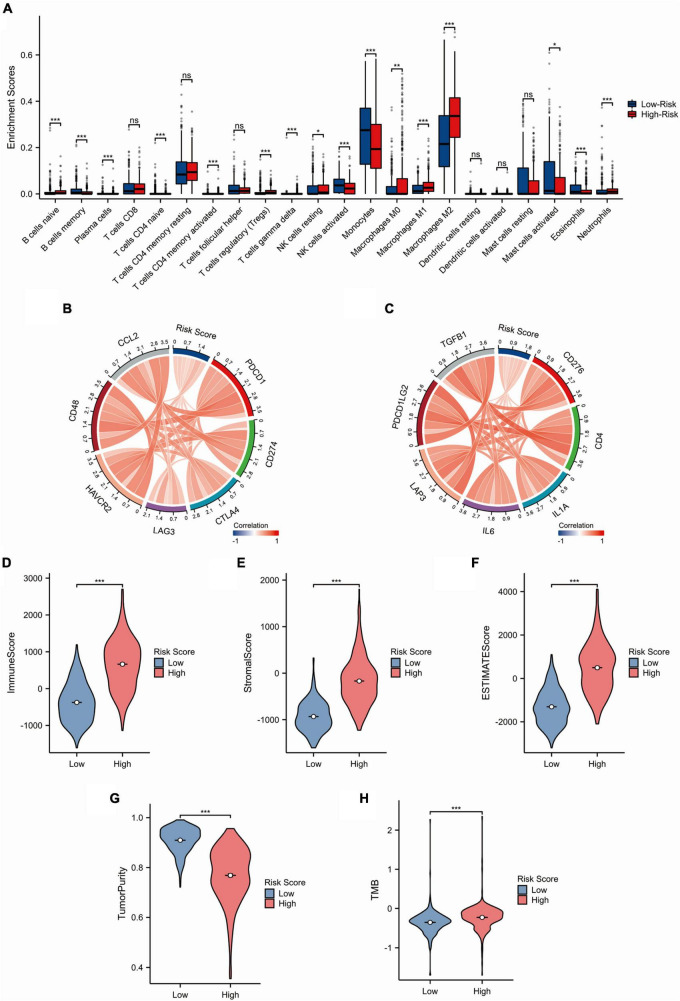
Immune-related analysis of the SRGs risk models in the TCGA cohort. **(A)** Comparison of immune cell infiltration between low- and high-risk groups. **(B,C)** Circular plots showing the correlation between the SRGs risk score and common immunotherapy-related molecules. **(D–H)** Comparison of immune-related scores (Immune score, Stromal score, ESTIMATE score, tumor purity and TMB) between high- and low-risk groups. (*p < 0.05; **p < 0.01; ***p < 0.001; ns, not significant).

We analyzed the infiltration extent of 22 immune cells in glioma patients with low- and high-risk scores in the TCGA dataset. The CIBERSORT analysis showed that the levels of macrophage (M0, M1, and M2), neutrophils, T cells regulatory (Tregs), T cells gamma delta, B-cell naive, NK cells resting, and T cells CD4 memory activated infiltration were higher in the high-risk group than in the low-risk group, whereas the proportion of NK cell activation, monocytes, B-cell memory, T-cell CD4 naive, plasma cells, mast cells activated, and eosinophils was higher in the low-risk group than in the high-risk group (Figure 4A). In addition, we investigated the relationship between expression of checkpoint genes and the SRGs risk scores. The results showed that the expression of 14 clinically common immune checkpoint genes was positively correlated with the risk scores (Figures 4B, C). Next, we analyzed immune-related parameters between low and high risk groups in the TCGA dataset. The results showed that immune score, stromal score, ESTIMATE score, and TMB were positively correlated with risk scores, while tumor purity was negatively correlated with risk score (Figures 4D–H). Furthermore, we investigated the relationship of VAMP5 and VAMP2 expression with the immune-related parameters. High expression of VAMP5 was positively correlated with immune score, stoma score and estimate score, while high expression of VAMP2 was negatively correlated with these scores (Supplementary Figure 2). These results suggested that the SRGs risk scores were associated with immune microenvironment of gliomas.

### 3.5 Construction of a prognostic nomogram containing the SRGs risk scores

**FIGURE 5:**
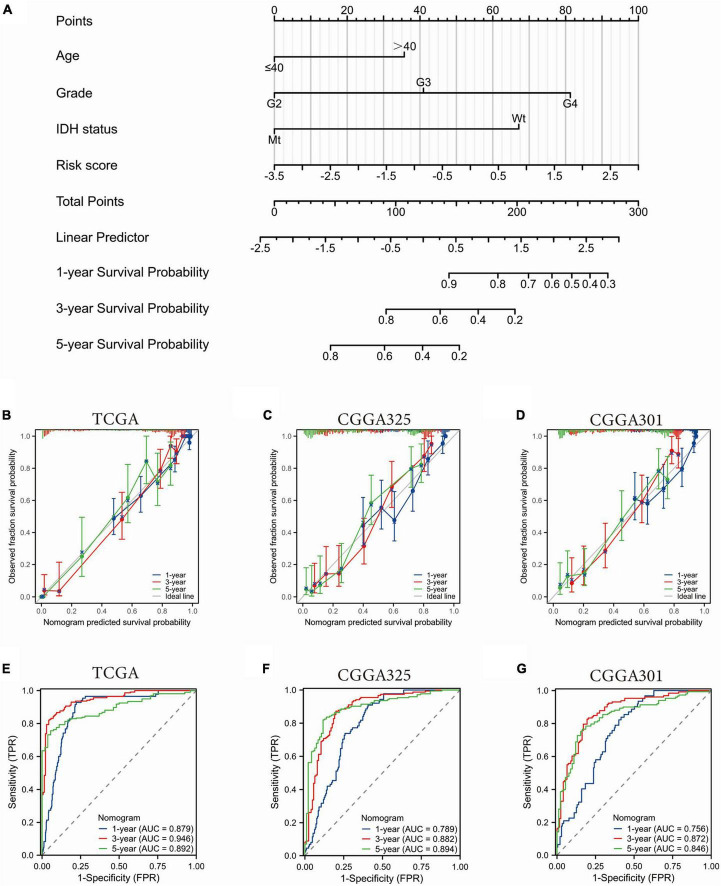
Construction and validation of a nomogram based on the SGRs risk scores and clinicopathological factors. **(A)** Prognostic profiles of 1-, 3-, and 5-year OS in the TCGA glioma dataset. **(B–D)** Calibration curves to evaluate the nomogram at 1, 3, and 5 years. The 45-degree gray line represents perfect alignment. **(E–G)** Time dependent ROC curve to evaluate the nomogram at 1, 3, and 5 years.

Univariate and multivariate COX regression analysis of common clinicopathological factors and the SRGs risk scores revealed that age, WHO grade, IDH mutation status, and the SRGs risk scores were independent prognostic factors (Supplementary Figure 3). In order to assess the prognostic predictive value of these independent factors, we constructed a nomogram by integrating the prognostic factors that met the proportional risk test into a line plot model. In the nomogram model, each of the above parameters was assigned a score according to a straight line drawn upward. The survival probability at 1, 3, and 5 years was estimated by a descending line from the total score line to the outcome line (Figure 5A and Supplementary Figure 4). The calibration curves showed good agreement between the nomogram predictions and the actual probabilities (Figures 5B–D). Additionally, the time-dependent ROC curves showed that our nomogram performed well in both the TCGA dataset and the CGGA datasets. In the TCGA cohort, the nomogram showed high predictive power for 1-, 3-, and 5-year OS with an AUC value of 0.879, 0.946, and 0.892, respectively (Figure 5E). The C-index of the nomogram was 0.852 (0.840–0.864). The predictive reliability of this nomogram was further validated in the CGGA325 and CGGA301 datasets (Figures 5F, G). These data suggested that the nomogram containing the SRGs risk score was an effective tool for predicting the prognosis of glioma patients.

### 3.6 Validation of the alteration of VAMP2 and VAMP5 in gliomas

**FIGURE 6:**
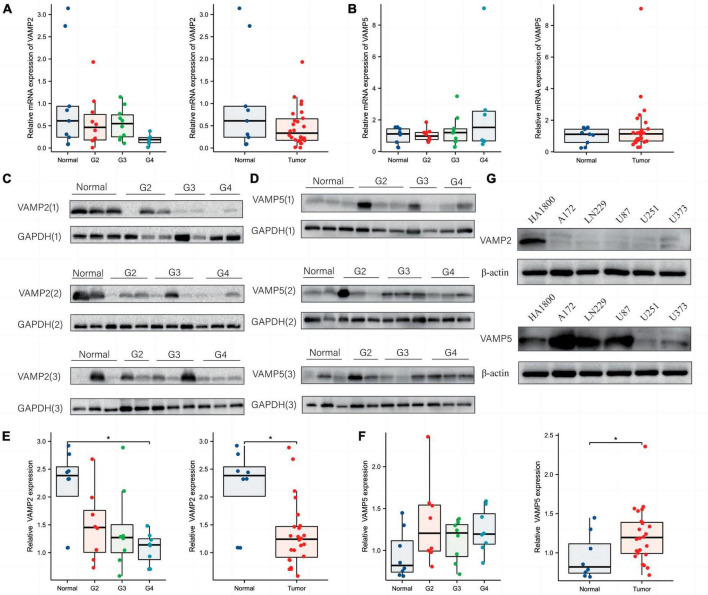
Validation of the changes of VAMP2 and VAMP5 in glioma specimens and cell lines. **(A,B)** qRT-PCR was used to quantify the changes of VAMP2 and VAMP5 at mRNA levels in WHO 2-4 gliomas and normal brain tissues. **(C–F)** Western blot analysis of the changes of VAMP2 and VAMP5 at protein levels in WHO 2-4 gliomas and normal brain tissues. Each band was derived from a subject. Three panels of blot images for VAMP2 and VAMP5 from all specimens were shown in **(C,D)**, respectively. Quantification graphs were shown in **(E)** (VAMP2) and **(F)** (VAMP5), respectively. **(G)** Western blot detection of VAMP2 and VAMP5 in the indicated glioma cell lines and a normal human astrocyte line (HA1800). (*p < 0.05).

We next validated the changes of VAMP2 and VAMP5 at both mRNA and protein levels in glioma specimens and cell lines stored in our institute. QRT-PCR assay showed that VAMP2 mRNA was gradually decreased with the increase of WHO grades, and that it reached the lowest level in WHO 4 gliomas compared to normal tissues (Figure 6A), while VAMP5 mRNA showed the opposite changes (Figure 6B). It should be noted that these differences were not statistically significant. Western blot analysis showed that VAMP2 protein level was also gradually decreased with the increase of WHO grades, and that there was a significant difference in the VAMP2 protein between glioma and normal tissues, which was mainly based on WHO 4 gliomas (Figures 6C, E). VAMP5 protein level showed an insignificant increase in different grades of gliomas, but it was significantly higher in glioma tissues than in normal tissues (Figures 6D, F). In addition, we found low expression of VAMP2 and high expression of VAMP5 in different glioma cell lines (U87, A172, LN229, U251, U373) compared to a human astrocyte line (HA1800) (Figure 6G). These results demonstrated the alteration of VAMP2 and VAMP5 in glioma specimens and cell lines, supporting the bioinformatics findings obtained from the public datasets.

## 4 Discussion

Tumor microenvironment (TME), a key factor in regulating glioma progression, can directly influence tumor growth and proliferation (Yin et al., 2019). It has been suggested that neurons and glioma cells are abnormally associated by chemical synapses, and neuronal activity can influence the malignant progression of gliomas (Gillespie and Monje, 2018). Gliomas are also capable of generating electrical synapses with the surrounding neural tissue, and this communication drives tumor cell growth and migration (Zahalka and Frenette, 2020). There are 36 members in the SNARE family (Bermingham and Blakely, 2016), and these genes are involved in the fusion of synaptic vesicles, which are required for neurotransmitter release into the synaptic cleft. Here we found that VAMP2 and VAMP5 were two key genes in the SNARE family that affected the prognosis of glioma patients. A recent study reported that SNAP25 inhibited glioma cell proliferation, migration and invasion by regulating the metabolism of glutamate (Huang et al., 2021). Therefore, VAMP2 and VAMP5 may play an important role in the progression of gliomas.

In most cases, biomarkers obtained by bioinformatic analysis are not validated by clinical samples, which limits the clinical application of new biomarkers. In this study, we identified VAMP2 and VAMP5 as two SRGs signature genes affecting the prognosis in both the training and validation sets. More importantly, we verified the changes of VAMP2 and VAMP5 at both mRNA and protein levels in different grades of gliomas collected from our hospital. Low VAMP2 and high VAMP5 were found in WHO 4 gliomas and cell lines. However, due to the lack of follow-up data in our cohort, we did not verify the relationship between VAMP2 and VAMP5 and the overall survival rate of glioma patients.

We constructed a risk model based on the expression of VAMP2 and VAMP5 in gliomas. Enrichment analysis on the DEGs between low- and high-risk patients showed that pathways that promote tumor metastasis and immune invasion were enriched in high-risk group, whereas pathways that inhibit synaptic transition and neuronal activity were enriched in low-risk group. The latter was agreed with the fact that VAMP2 and VAMP5 belongs to the SNARE family. SNARE proteins have been suggested to mediate the secretion of proteins and hormones, as well as phagocytosis and transport of pathogens by the immune system (Cuddy et al., 2019). Bioinformatic analysis of some immune-related genes has shown that VAMP5 is associated with immune infiltration in some tumor types (Xiong et al., 2020). We further explored the differences between low- and high-risk group in immune cell infiltration. The proportion of macrophages, neutrophils and B-cell naïve were higher in high-risk group, whereas the proportion NK cell, CD4 T cell and B-cell memory were higher in low-risk group.

The infiltration of macrophages and neutrophils in gliomas promoted angiogenesis, suppressed the immune system, remodeled the extracellular matrix, and promoted glioma growth and invasion (Rahbar et al., 2016; Massara et al., 2017; Li et al., 2023), which may contribute to the poor prognosis of high-risk patients. NK cells exert cytolytic activity by secreting cytokines to kill susceptible target cells (Loftus et al., 2018). Previous studies have found that naive CD4^+^ T cells, activated mast cells and monocytes are protective factors for the prognosis of glioma patients (Luo et al., 2021). Thus, the infiltration of these immune cells might be responsible for the better prognosis in low-risk group. Immune-related score analysis showed that the immune score was higher in the high-risk group, supporting TME imbalance and abnormal aggregation of immune cells in the high-risk group. In addition, the expression levels of 13 immune checkpoint genes were positively correlated with risk scores, suggesting that patients in the high-risk group could benefit more from treatment with immune checkpoint inhibitors.

Our study confirmed that the expression levels of VAMP2 and VAMP5 genes were significantly associated with survival time, WHO classification, IDH, and co-deletion status of 1p19q in glioma patients. VAMP2, also known as synaptic Brevin-2 (SYB2), is a key synaptic vesicle fusion protein (Banks et al., 2020), and it plays a critical role in the formation of the SNARE complex (Martineau et al., 2017). VAMP5, as a crucial molecule mediating vesicle-mediated transport and Golgi to plasma membrane protein transport processes, has a role in promoting tumor progression, but is less studied. We found that age, WHO grade, IDH mutation status, and the SRGs risk scores were independent factors in predicting the prognosis of glioma patients, a nomogram was established based on these factors. Both calibration and ROC curves confirmed that the nomogram containing the SRG score was an effective tool for predicting patients’ prognosis.

There are still some limitations for this study. Firstly, though the risk model based on the key SNARE proteins was constructed based on TCGA glioma dataset and was validated in CGGA and our glioma cohorts, further study with a larger sample size is needed to confirm the effectiveness and accuracy of the risk model. Secondly, we identified VAMP2 and VAMP5 as two key SNARE proteins affecting the prognoses of gliomas, but the lack of experimental evidences in glioma cells or models limits the interpretation of this finding. Thirdly, we found that the SRGs risk score was closely related to the TME of gliomas, e.g., immune cell infiltration, neuronal activity. Thus, co-culture systems of neurons or immune cells with glioma cells are required to reveal the roles of VAMP2 and VAMP5 in the TME of gliomas.

## 5 Conclusion

In this study, we constructed a novel risk model for predicting the prognosis and immune microenvironment of glioma patients based on the expression of SNARE proteins VAMP2 and VAMP5. The model showed satisfactory predictive performance in both training and validation cohorts. There were pronounced differences in the pathways of tumor proliferation and metastasis, and in immune cell infiltration and neuronal activity of the TME between low- and high-risk glioma patients. In addition, we constructed a nomogram plot based on the SRG risk score and common clinicopathological features, which was proved to be excellent in predicting the prognosis of glioma patients. Furthermore, we verified the changes of VAMP2 and VAMP5 in glioma specimens and cell lines stored in our institute. In conclusion, this study provides a useful tool for clinically predicting the prognosis and responsiveness to immunotherapy in gliomas.

## Data availability statement

The raw data supporting the conclusions of this article will be made available by the authors, without undue reservation.

## Ethics statement

The studies involving human specimens were approved by the Ethics Committee of Affiliated Hospital of Xuzhou Medical University. The studies were conducted in accordance with the local legislation and institutional requirements. The participants provided their written informed consent to participate in this study.

## Author contributions

LY: Writing – review and editing, Data curation, Investigation. YX: Data curation, Writing – original draft, Investigation, JY: Funding acquisition, Methodology, Writing – review and editing. HC: Formal analysis, Software, Writing – review and editing. WX: Formal analysis, Software, Writing – review and editing. YW: Software, Writing – review and editing. DJ: Conceptualization, Funding acquisition, Writing – review and editing. SG: Conceptualization, Funding acquisition, Writing – review and editing.

## Footnotes

1
https://cancergenome.nih.gov

2
https://www.ncbi.nlm.nih.gov/gds/

3
http://www.cgga.org.cn
