# Supplementary figures and images for: Construction and validation of a risk model based on the key SNARE proteins to predict the prognosis and immune microenvironment of gliomas
Source: Front Mol Neurosci. 2023 Dec 5;16:1304224. doi: 10.3389/fnmol.2023.1304224 (PMC10728289; doi:10.3389/fnmol.2023.1304224)

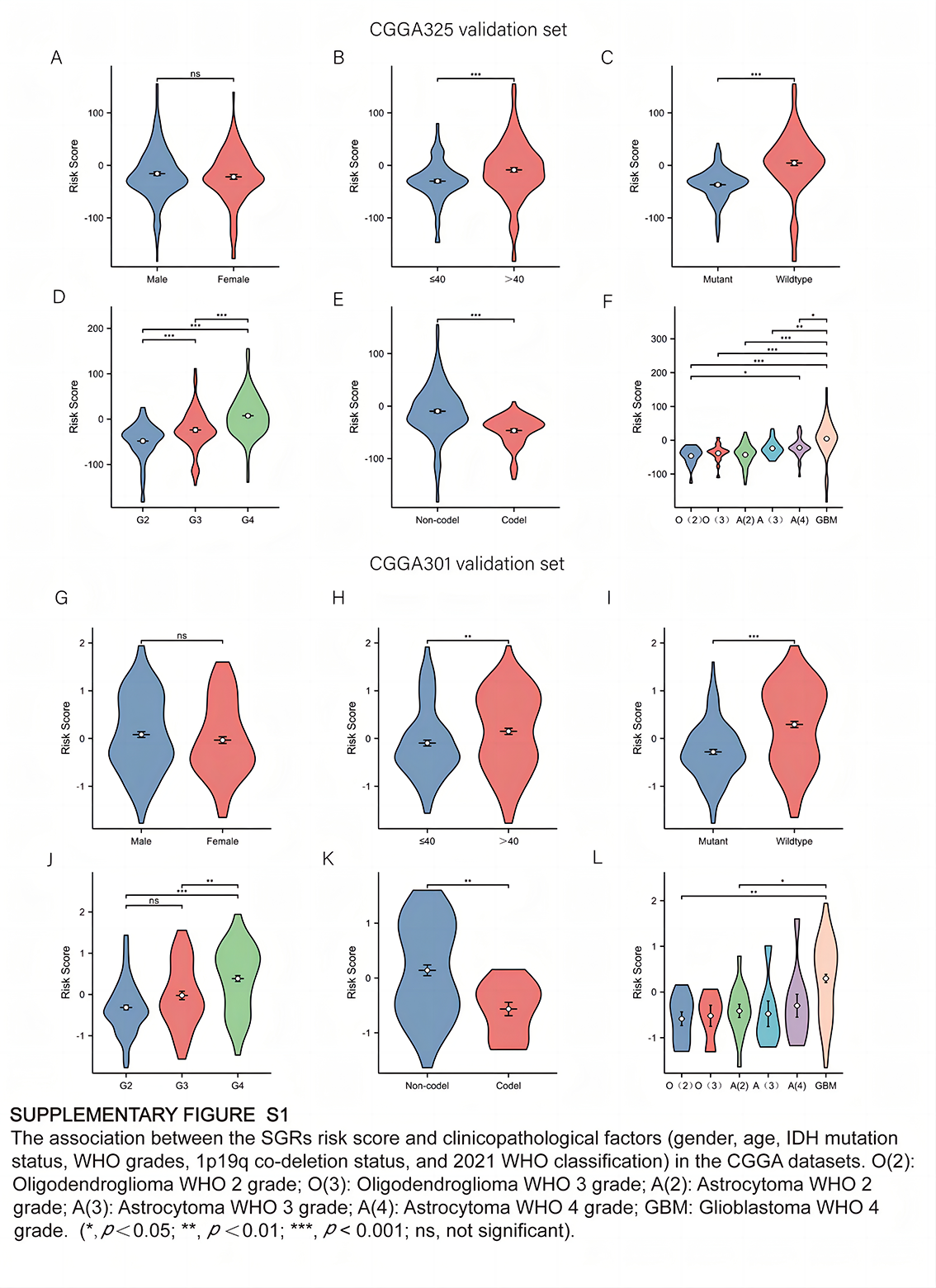

Supplement: Supplementary file 1 [file Image_1.TIF]

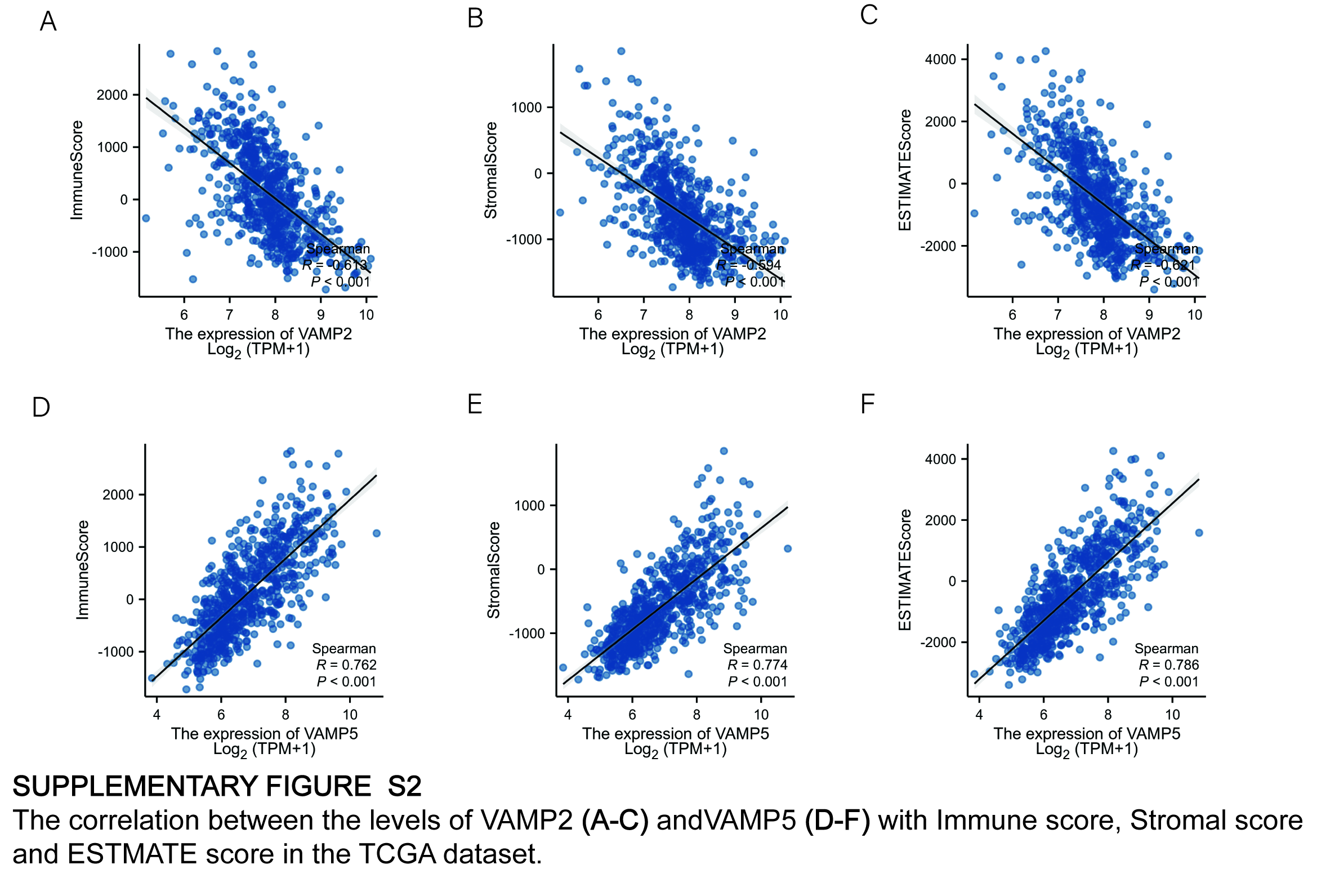

Supplement: Supplementary file 2 [file Image_2.TIF]

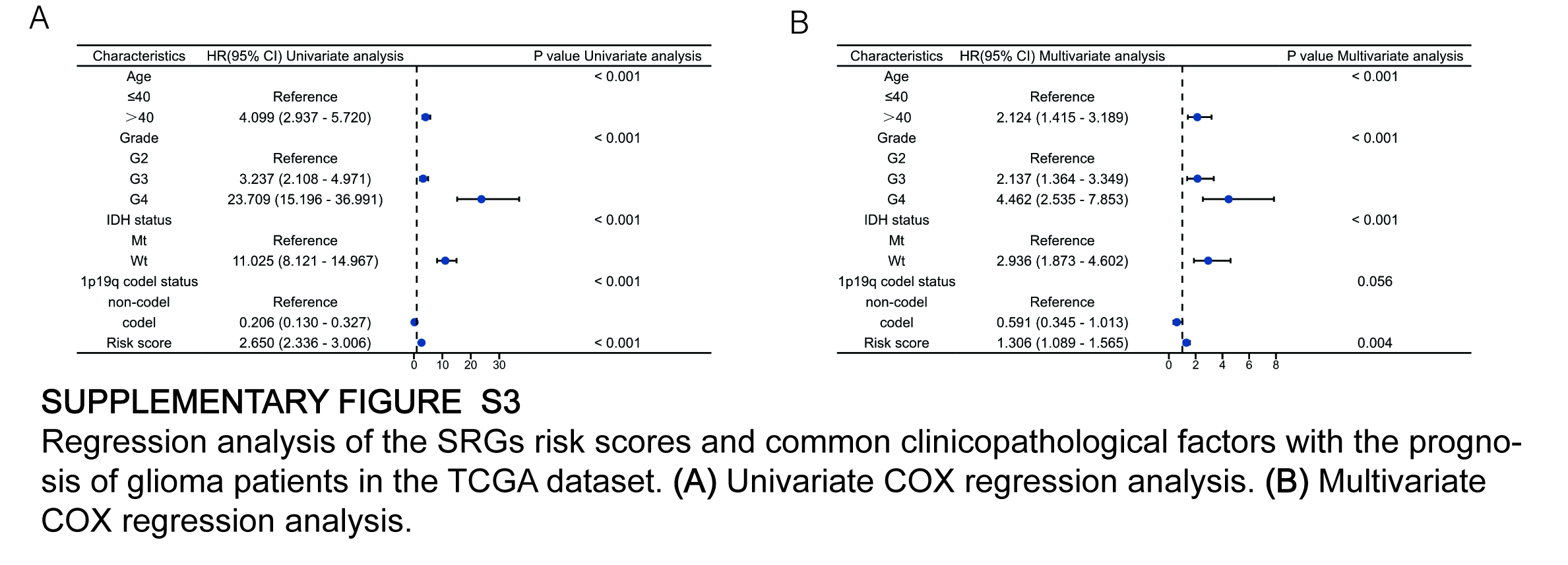

Supplement: Supplementary file 3 [file Image_3.TIF]

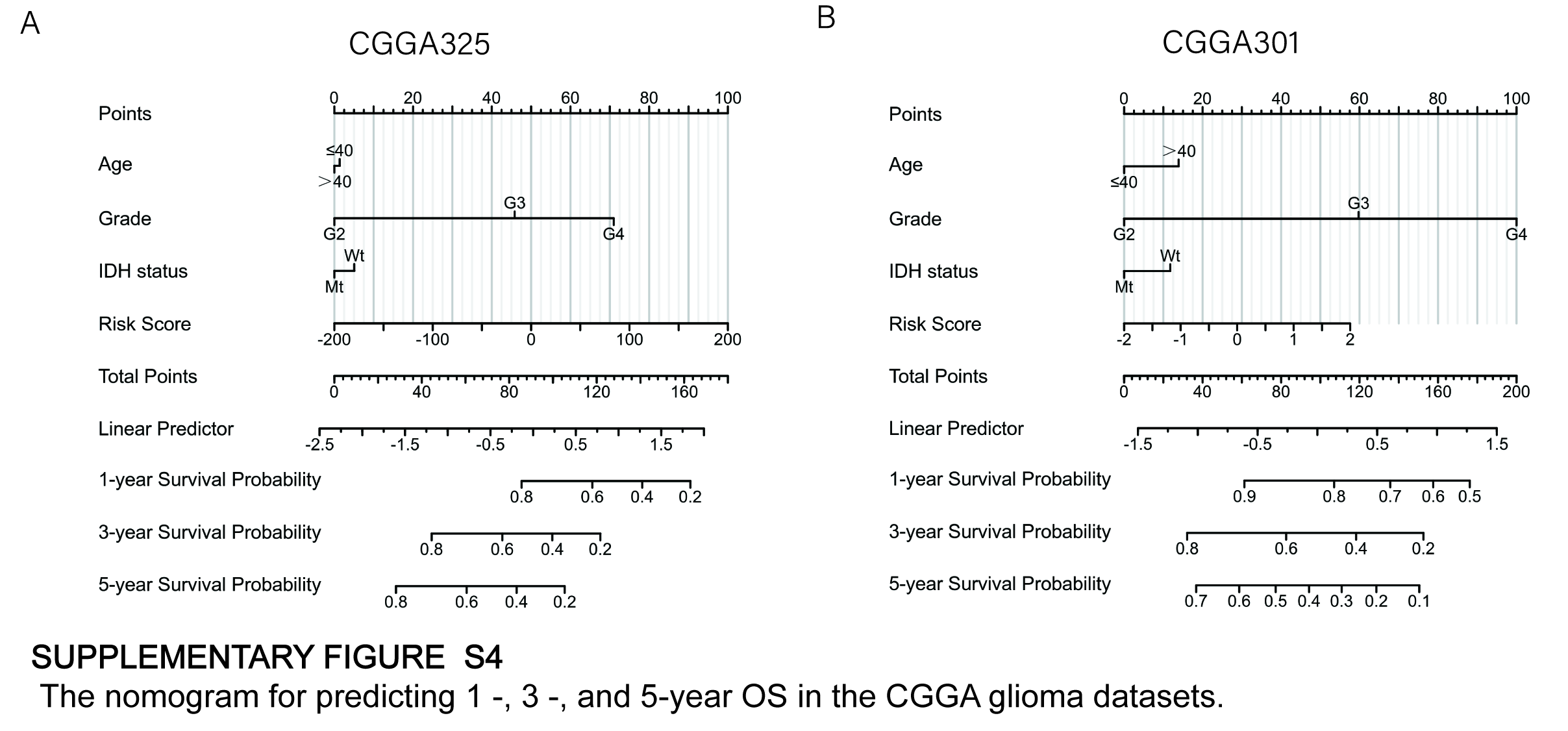

Supplement: Supplementary file 4 [file Image_4.TIF]
